# Transformer-based Deep Learning Models with Shape Guidance for Predicting Breast Cancer in Mammography Images

**DOI:** 10.1007/s10278-025-01773-3

**Published:** 2025-12-19

**Authors:** Kengo Takahashi, Yuwen Zeng, Zhang Zhang, Kei Ichiji, Takuma Usuzaki, Ryusei Inamori, Haoyang Liu, Noriyasu Homma

**Affiliations:** 1 Department of Radiological Imaging and Informatics, Tohoku University Graduate School of Medicine, 2-1 Seiryo-Machi, Aoba-Ku, Sendai, Miyagi 980-8575 Japan; 2 Tohoku University Advanced Institute of So-Go-Chi (Convergence Knowledge) Informatics, 2-1-1 Katahira, Aoba-Ku, Sendai, Miyagi 980-0812 Japan; 3 Center for Data-Driven Science and Artificial Intelligence, Tohoku University, 41 Kawauchi, Aoba-Ku, Sendai, Miyagi 980-8576 Japan; 4 Department of Diagnostic Radiology, Tohoku University Hospital, 1-1 Seiryo-Machi, Aoba-Ku, Sendai, 980-8574 Japan; 5 Department of Diagnostic Imaging , Tohoku University Graduate School of Medicine, 2-1 Seiryo-Machi, Aoba-Ku, Sendai, Miyagi 980-8575 Japan; 6 Intelligent Biomedical Systems Engineering Laboratory, Graduate School of Biomedical Engineering, Tohoku University, 2-1 Seiryo-Machi, Aoba-Ku, Sendai, Miyagi 980-8575 Japan

**Keywords:** Breast cancer, Mammography image, Vision Transformer, Swin Transformer, Shape guidance

## Abstract

Recent breast cancer research has investigated shape-based attention guidance in Vision Transformer (ViT) models, focusing on anatomical structures and the heterogeneity surrounding tumors. However, few studies have clarified the optimal transformer encoder layer stage for applying attention guidance. Our study aimed to evaluate the effectiveness of shape-guidance strategies by varying the combinations of encoder layers that guide attention to breast structures and by comparing the proposed models with conventional models. For the shape-guidance strategy, we applied breast masks to the attention mechanism to emphasize spatial dependencies and enhance the learning of positional relationships within breast anatomy. We then compared the representative models—Masked Transformer models that demonstrated the best performance across layer combinations—with the conventional ResNet50, ViT, and SwinT V2. In our study, a total of 2,436 publicly available mammography images from the Chinese Mammography Database via The Cancer Imaging Archive were analyzed. Three-fold cross-validation was employed, with a patient-wise split of 70% for training and 30% for validation. Model performance on differentiating breast cancer from non-cancer images was assessed by the area under the receiver-operating characteristic curve (AUROC). The results showed that applying masks at the Shallow and Deep stages gave the highest AUROC for Masked ViT. The Masked ViT achieved an AUROC of 0.885 [95% confidence interval: 0.849–0.918], a sensitivity of 0.876, and a specificity of 0.802, outperforming all other conventional models. These results indicate that incorporating mask guidance into particular Transformer encoders promotes representation learning, highlighting their potential as decision-support tools in breast cancer diagnosis.

**Supplementary Information:**

The online version contains supplementary material available at https://doi.org/10.1007/s10278-025-01773-3.

## Introduction

Breast cancer is the most common cancer among women worldwide [1]. In 2022, over 2.3 million cases were diagnosed and 670,000 patients died from breast cancer [2]. Screening mammography is the only examination that has been shown to reduce breast cancer mortality, reportedly by as much as 30% via early detection [3, 4]. Although mammography is widely recognized as a useful tool for diagnosing breast cancer, false-negative and false-positive recall rates remain high in patients with dense breast tissue [5].

Recently, artificial intelligence (AI) has emerged as a promising quantitative tool for computer-aided diagnosis (CAD) in mammographic screening [6]. Many AI studies focus on regions of interest (ROI) selected based on expert knowledge [7–10]. Limiting training to these targeted regions enables the performance assessment of these AI models to identify cancer-related structures. In addition, this approach reduces the computational burden and can leverage models pre-trained on a large number of images, since mammography images are typically high-resolution [11]. However, relying solely on ROI images may lead to the loss of important structural and contextual information [12]. In fact, parenchymal phenotypes based on radiomic features across the entire breast were associated with increased breast cancer risk [13], and non-mammary zone features are also known to contribute to missed diagnoses by AI [14]. Radiologists assess the risk of breast cancer by considering the overall pattern and characteristics of breast tissue in mammography images [15]. Thus, capturing both lesions and their surrounding anatomical context from entire mammography images is essential for developing AI models for clinical use [16].

CNNs (Convolutional neural networks) have inherent limitations in capturing global context. In contrast, the Vision Transformer (ViT) architecture has attracted much attention for its outstanding ability to capture long-range dependencies [17]. Using an attention mechanism, Transformer-based models are capable of, for example, comprehensively capturing the positions of words in a sentence. However, its attention mechanism also risks introducing irrelevant correlations that hinder the capture of meaningful patterns [18]. Recent studies have therefore incorporated shape guidance to improve visual attention. Zhiheng et al. demonstrated the utility of incorporating learnable causal masks into the Segment Anything Model for anatomical segmentation tasks [19]. Ma et al. showed the effectiveness of eye-gaze-guided ViT for mammography and chest X-rays, integrating physicians’ visual attention into attention maps [20]. Hatamizadeh et al. proposed the Vision Retentive Network [21], which learns spatial relationships between successive horizontal and vertical positions by guiding the attention map based on a specific formulation. Their network captured both short and long-range spatial dependencies effectively. Overall, these methods incorporating refined attention showed improved feature extraction and interpretability.

However, few studies have examined the optimal transformer encoder layer for guiding the attention. Additionally, comparisons with conventional algorithms are clinically important to understand how particular strategies differently affect diagnostic sensitivity and specificity in breast cancer detection [22]. Our study aimed to evaluate the effectiveness of shape-guidance strategies by varying the combinations of encoder layers that guide attention to breast structures.

## Materials and Methods

### Data Collection

All images were obtained from the Chinese Mammography Database (CMMD), in accordance with The Cancer Imaging Archive data usage policy [23]. The retrospective data collections were approved by the institutional review boards of the Sun Yat-sen University Cancer Center and Nanhai Affiliated Hospital of Southern Medical University. The database contains 5,202 mammography images of 1,775 patients with a biopsy-confirmed benign or malignant tumor. Patients were examined between July 2012 and January 2016.

The recently available TOMPEI-CMMD dataset is an enhanced version of the original CMMD dataset, using the same patient cohort with mammography but providing annotations and lesion-level diagnostic labels [24]. The enhanced dataset consists of 2,601 mediolateral oblique (MLO) views from the original CMMD. All radiological findings (i.e., mass, calcification, fibroadenoma, distortion, focal asymmetric density, and lipoma) were labeled by a radiologist with over 20 years of experience, based on the final outcomes of benign/malignant diagnosis in the original CMMD dataset. Segmentation was conducted by five radiological technologists based on the radiologist’s evaluations. Out of the 2,601 images, the following were excluded: phyllodes tumors (N = 6), neurofibromatosis (N = 2), lymphedema (N = 1), central venous ports (N = 8), visible foreign bodies (N = 5), image artifacts hindering diagnosis (N = 3), and breast cancer cases with no identifiable lesions (N = 140). The dataset used in the present study comprised a total of 2,436 images: 1,167 images with breast cancer and 1269 images without breast cancer (215 images with benign lesions, 1054 breasts with normal breast tissue).

Images were obtained on a GE Senographe DS mammography system (GE Healthcare, United States) and a Siemens Mammomat Inspiration mammography system (Siemens Healthineers, Germany). All mammography images were originally acquired at a resolution of 2294 × 1914 pixels. Although images are typically resized to de facto standard resolutions (e.g., 224 × 224 or 512 × 512 pixels) before being inputted into AI models, downscaling mammography images would result in a substantial loss of diagnostically relevant information, as has been previously demonstrated [25]. A preliminary experiment we conducted also confirmed that model performance decreases with lower image resolution (Appendix 1). Therefore, to preserve as much anatomical detail as possible, we applied a padding-based preprocessing and subsequently resized the images to 2048 × 2048 pixels using LANCZOS resampling with the PILLOW library (Appendix 2).

### ViT Architecture and Mask Guidance

**Fig. 1:**
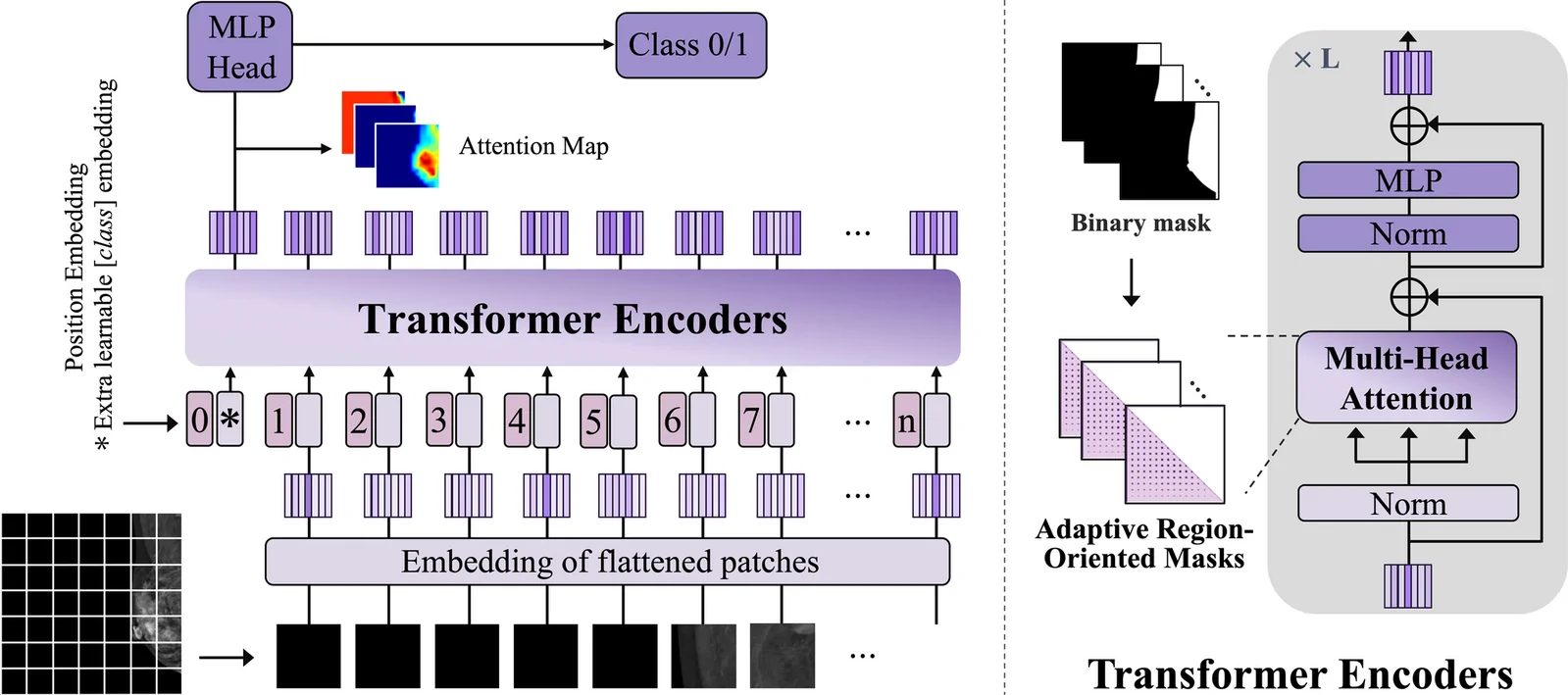
Architectural overview of Masked ViT: Mammography images are cut into patches and each is linearly embedded before adding position embeddings and feeding the sequence of vectors to encoders. Causal masks of breast structures are applied to the multi-head attention mechanism in the encoders.

We added a learnable [CLS] classification token to aggregate the global information of the entire image (Fig. 1). This token is used in both training and classification. The success of the Transformer-based models, such as ViT, is due to their self-attention (SA) mechanism, which learns the relationships of a single token with respect to all other tokens in the sequence [17, 26]. Long-range dependencies can be captured utilizing the SA mechanism, which is implemented by defining three learnable weight matrices to transform the input into Queries (${\mathbf{W}}_{{\boldsymbol{Q}}} \in {\mathbb{R}}^{d\times {d}_{k}}$), Keys (${\mathbf{W}}_{{\boldsymbol{K}}} \in {\mathbb{R}}^{d\times {d}_{k}}$), and Values (${\mathbf{W}}_{{\boldsymbol{V}}} \in {\mathbb{R}}^{d\times {d}_{v}}$), where ${d}_{k}= {d}_{v}$. Given an input sequence $\mathbf{X} \in {\mathbb{R}}^{n\times d}$, where n is the sequence length, the projections are defined as $\mathbf{Q}=\mathbf{X}{\mathbf{W}}_{{\boldsymbol{Q}}}$, $\mathbf{K}=\mathbf{X}{\mathbf{W}}_{{\boldsymbol{K}}}$, and $\mathbf{V}=\mathbf{X}{\mathbf{W}}_{{\boldsymbol{V}}}$,. The output ($\mathbf{Z} \in {\mathbb{R}}^{n\times {d}_{v}}$) of the SA layer is then defined as:

1 $${\mathbf{Z}}= {\mathbf{SA}}(\mathbf{Q}, \mathbf{K}, \mathbf{V})={\mathbf{softmax}}\left(\frac{{\mathbf{Q}\mathbf{K}}^{\mathrm{T}}}{\sqrt{{d}_{k}}} \right)\mathbf{V}.$$

We can optionally mask the SA blocks used in the decoder to prevent attending to subsequent future tokens. The masked SA (MSA) using a mask ($\mathbf{M} \in {\mathbb{R}}^{n\times n}$) is given by:

2 $$\begin{array}{c}{\mathbf{Z}}= {\mathbf{M}}{\mathbf{SA}}\left(\mathbf{Q}, \mathbf{K}, \mathbf{V}\right)={\mathbf{softmax}}\left(\mathbf{A}\right)\mathbf{V},\mathbf{A}=\frac{{\mathbf{Q}\mathbf{K}}^{\mathrm{T}}}{\sqrt{{d}_{k}}}\\ {\mathrm{A}}_{\mathrm{ij}}=\left\{\begin{array}{c}{\mathrm{A}}_{\mathrm{ij}}, {\mathrm{M}}_{\mathrm{ij}}>0\\ -\infty , {\mathrm{M}}_{\mathrm{ij}}=0\end{array}\right.\end{array},$$

where $\mathbf{M}$ is typically a lower-triangular matrix. Multi-head self-attention (MHSA) performs parallel computation of multiple SAs, enabling the model to capture information across different positions [17, 26]. The number of attention heads corresponds to the number of SAs in MHSA, resulting in the same number of inference visualization images. The MHSA is defined as:

3 $${\mathbf{MHSA}}\left(\mathbf{Q}, \mathbf{K}, \mathbf{V}\right)= \, \left[{\mathbf{Z}}_{0},{\mathbf{Z}}_{1},\dots ,{\mathbf{Z}}_{h-2},{\mathbf{Z}}_{h-1}\right]{\mathbf{W}}_{O},$$

where h denotes the total number of heads in an MHSA block. ${\mathbf{W}}_{O}\in {\mathbb{R}}^{h{\bullet d}_{v}\times d}$ is a weight matrix for linear projection, where d is the entity embedding dimension. In our study, we used 12 attention heads with a 768-dimensional embedding space, giving 64 dimensions per head to capture positional relationships.

**Fig. 2:**
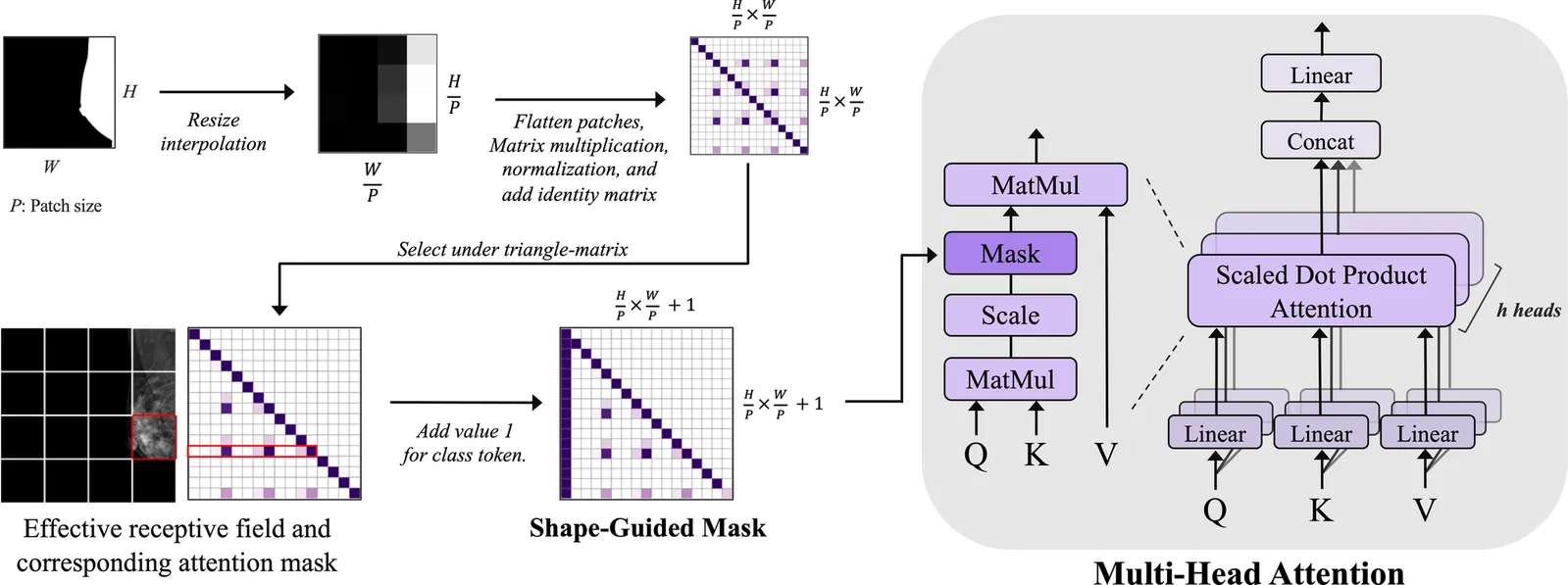
Mechanism for creating shape-guided masks: While a 4 × 4 patch grid is shown here for illustrative purposes, the actual model used 16 × 16 patches to capture finer spatial relationships. Cell opacity represents the decay of attention strength based on the distance between the highlighted and colored cells.

In natural language models, a weighted mask $\mathbf{M}$ decreasing toward the lower-left corner is used to prioritize closer token relationships. In natural image models, Sun et al. explored a mask weighted by 2D Manhattan distance for directing strong attention to neighboring patches [27]. Inspired by contextual modeling in Transformer processing—where sequential dependencies between words or patches are explicitly learned through a weighted mask—we applied a similar concept to spatial relationships in medical images. In our study, breast segmentation masks were generated for regions containing at least one pixel intensity value to help the ViT model focus on anatomical structures. Then, to adjust the mask to match the dimensions of the MHSA weights for breast shape, the following steps were applied to each breast segmentation mask: resizing with BICUBIC interpolation, flattening as patches, calculation of normalized inner products, and selecting the lower-triangular matrix (Fig. 2). Finally, we set the attention scores of masked-out regions to negative infinity before the softmax step, ensuring that they receive zero attention weight.

### SwinT V2 Architecture and Mask Guidance

The Swin Transformer introduces a hierarchical architecture to the basic Transformer unit through window-based attention, enabling effective recognition of objects at different scales. SwinT does not employ a [CLS] token; instead, the final prediction is obtained by applying global average pooling across the final feature maps. The output $\mathbf{Z} \in {\mathbb{R}}^{{M}^{2}\times d}$ from SA within a local window of size M×M is calculated as:

4 $${\mathbf{Z}}= {\mathbf{SA}}(\mathbf{Q}, \mathbf{K}, \mathbf{V})={\mathbf{softmax}}\left(\frac{{\mathbf{Q}\mathbf{K}}^{\mathrm{T}}}{\sqrt{d}}+{\boldsymbol{B}}\right)\mathbf{V},$$

where ${\boldsymbol{B}} \in {\mathbb{R}}^{{M}^{2}\times {M}^{2}}$ is the relative position bias term for each head. The matrices $\mathbf{Q},\mathbf{K},\mathbf{V} \in {\mathbb{R}}^{{M}^{2}\times d}$ represent the query, key, and value, respectively; d is the query/key dimension, and ${M}^{2}$ is the number of patches in a window. The learnable parameters are only defined for (2 M−1) × (2 M−1) discrete relative positions, and the relative position bias is parameterized as a bias matrix $\widehat{{\boldsymbol{B}}} \in {\mathbb{R}}^{(2M-1)\times (2M-1)}$. To prevent specific pixel pairs from skewing attention distributions, SwinT V2 adopts a scaled cosine attention mechanism:

5 $${\mathbf{SA}}\left({\boldsymbol{Q}},\boldsymbol{ }{\boldsymbol{K}},\boldsymbol{ }{\boldsymbol{V}}\right)= {\mathbf{softmax}}\left({\mathbf{Sim}}\left({\boldsymbol{Q}},\boldsymbol{ }{\boldsymbol{K}}\right)\right) {\boldsymbol{V}}={\mathbf{softmax}}\left({\mathbf{cos}}({\boldsymbol{Q}},\boldsymbol{ }{\boldsymbol{K}})\;\tau +\boldsymbol{ }{\boldsymbol{S}}\right) {\boldsymbol{V}},$$

where ${\boldsymbol{Q}},\boldsymbol{ }{\boldsymbol{K}},\boldsymbol{ }{\boldsymbol{V}}\boldsymbol{ }\in \boldsymbol{ }{\mathbb{R}}^{{M}^{2}\times d}$, ${\boldsymbol{S}}\boldsymbol{ }\in \boldsymbol{ }{\mathbb{R}}^{{M}^{2}\times {M}^{2}}$, and τ is a learnable scalar. The masked SA (MSA) is computed by incorporating the corresponding mask ($\mathbf{M} \in {\mathbb{R}}^{{M}^{2}\times {M}^{2}}$) during the calculation of window-based attention:

6 $$\begin{array}{c}{\mathbf{Z}}= {\mathbf{M}}{\mathbf{SA}}\left(\mathbf{Q}, \mathbf{K}, \mathbf{V}\right)={\mathbf{softmax}}\left(\mathbf{A}\right) {\boldsymbol{V}},\mathbf{A}={\mathbf{cos}}({\boldsymbol{Q}},\boldsymbol{ }{\boldsymbol{K}})\tau +\boldsymbol{ }{\boldsymbol{S}}\\ {\mathrm{A}}_{\mathrm{ij}}=\left\{\begin{array}{c}{\mathrm{A}}_{\mathrm{ij}}, {\mathrm{M}}_{\mathrm{ij}}>0\\ -\infty , {\mathrm{M}}_{\mathrm{ij}}=0\end{array}\right.\end{array},$$

where $\mathbf{M}$ is computed from the same spatial region as the attention window.

### Model Comparison

First, we evaluated model performance by applying shape-guided masks at three encoder stages: Shallow (L1–L4), Middle (L5–L8), and Deep (L9–L12). Three-fold cross-validation was employed, with a patient-wise split of 70% for training and 30% for validation data. Following the ablation study, the models that showed the highest performance in terms of the mean area under the receiver-operating characteristic curve (AUROC) across folds were selected as representative models for the shape guidance strategy. Specifically, we selected one representative model for the Masked Vision Transformer (Masked ViT) and one for the Masked Swin Transformer V2 (Masked SwinT V2).

Utilizing the same test dataset, we then compared the representative models with the following conventional models: Residual neural Network 50 (ResNet50) [28], standard SwinT V2 [29], and standard ViT [17]. The conventional ViT architecture is often compared to CNNs such as ResNet from both a historical and structural perspective due to its residual connections [30–32]. In addition, ResNet is a representative model used for breast cancer prediction [33] and shares architectural similarities with Transformer encoders. Swin Transformer (SwinT) computes attention within localized windows for multi-scale feature extraction [34]. SwinT V2 enhances the original SwinT by introducing scaled cosine attention and post-norm layers, preventing training saturation even with high-resolution images. Our preliminary experiments also showed that SwinT V2 outperformed SwinT (Appendix 3). Furthermore, previous research found SwinT V2 to be competitive with conventional CNNs for breast cancer detection in mammography images [22].

### Model Performance

Model performance was primarily evaluated using the AUROC and the area under the precision-recall curve (AUPRC). Additional metrics included accuracy (ACC), sensitivity (SEN), specificity (SPE), F1-score (F1), and Matthews correlation coefficient (MCC).

### Experimental Setup

We optimized a weighted cross-entropy (WCE) loss function to address class imbalance. The weights for the negative class $\left(W_{negative}\right)$ and positive class$\left(W_{positive}\right)$ were calculated as:

7 $${w}_{negative}= \frac{{N}_{positive}}{{N}_{negative}+ {N}_{positive}},$$

8 $${w}_{positive}= \frac{{N}_{negative}}{{N}_{negative}+ {N}_{positive}},$$

where ${N}_{negative}$ and ${N}_{positive}$ indicate the numbers of samples in the negative and positive classes, respectively. By assigning higher weights to the minority class, the WCE loss penalizes misclassifications of underrepresented samples more heavily. This approach balances the gradient contributions during optimization and mitigates the model’s bias toward the majority class. The weighting ratios (${w}_{negative}$, ${w}_{positive}$) applied in our study were as follows: Fold #1 (0.415, 0.585); Fold #2 (0.424, 0.576); Fold #3 (0.416, 0.584). The WCE loss function was optimized using the AdamW optimizer, and the batch size was 4. The initial learning rate was experimentally set to 1.0 × 10^–5^, and was reduced to 1.0 × 10^–6^ using the Cosine Annealing LR scheduler. Gaussian error linear unit (GELU) was used in the Transformer encoders as the activation function. Other hyperparameters were set as follows: patch size, 128 × 128; dimension of linear layers to project patches, 768; layers, 12; number of attention heads, 12.

We saved the model parameters corresponding to the lowest validation loss across 30 training epochs. AUROC and AUPRC values were reported with 95% confidence interval (95% CI), calculated using bootstrapping with 1,000 random resamples. We compared AUROC values between models using the DeLong test, with statistical significance defined as a p-value less than 0.05 (two-tailed). All models were implemented in PyTorch (version 2.5.1) with Python (version 3.10.12), using the Timm package (version 1.0.12) for architecture configuration.

## Results

Table 1 summarize the mean AUROC and AUPRC values across folds for Masked ViT and Masked SwinT V2, respectively. In Masked ViT, guidance configurations—Shallow, Shallow & Middle, Shallow & Deep, Shallow & Middle & Deep, Middle, Middle & Deep, and Deep—achieved higher AUROC values than the No-Mask baseline. In Masked SwinT V2, only the Shallow & Deep configuration outperformed the No-Mask model. For both Masked ViT and Masked SwinT V2, masking at the Shallow & Deep stages achieved the highest AUROC. Specifically, the Shallow & Deep configuration reached an AUROC of 0.885 (95% CI: 0.849–0.918) in Masked ViT and 0.879 (0.845–0.912) in Masked SwinT V2. These two types of models were therefore selected as representative models for the shape-guided strategy.

**Table 1:** Stage-wise ablation study

Shape-guided strategy			AUROC [95% CI]	
Shallow (L1–L4)	Middle (L5–L8)	Deep (L9–L12)	Masked ViT	Masked SwinT V2
-	-	-	0.864 [0.827–0.899]	0.877 [0.842–0.911]
✓	-	-	0.866 [0.827–0.903]	0.864 [0.826–0.900]
✓	✓	-	0.882 [0.845–0.915]	0.873 [0.836–0.909]
✓	-	✓	**0.885** **[0.849–0.918]**	**0.879** **[0.845–0.912]**
✓	✓	✓	0.865 [0.826–0.902]	0.876 [0.840–0.911]
-	✓	-	0.875 [0.840–0.908]	0.875 [0.840–0.908]
-	✓	✓	0.870 [0.833–0.906]	0.870 [0.833–0.906]
-	-	✓	0.874 [0.838–0.909]	0.871 [0.833–0.907]

Abbreviations: 95% CI: 95% confidence interval; AUC-ROC: area under the curve of receiver-operating characteristics

**Fig. 3:**
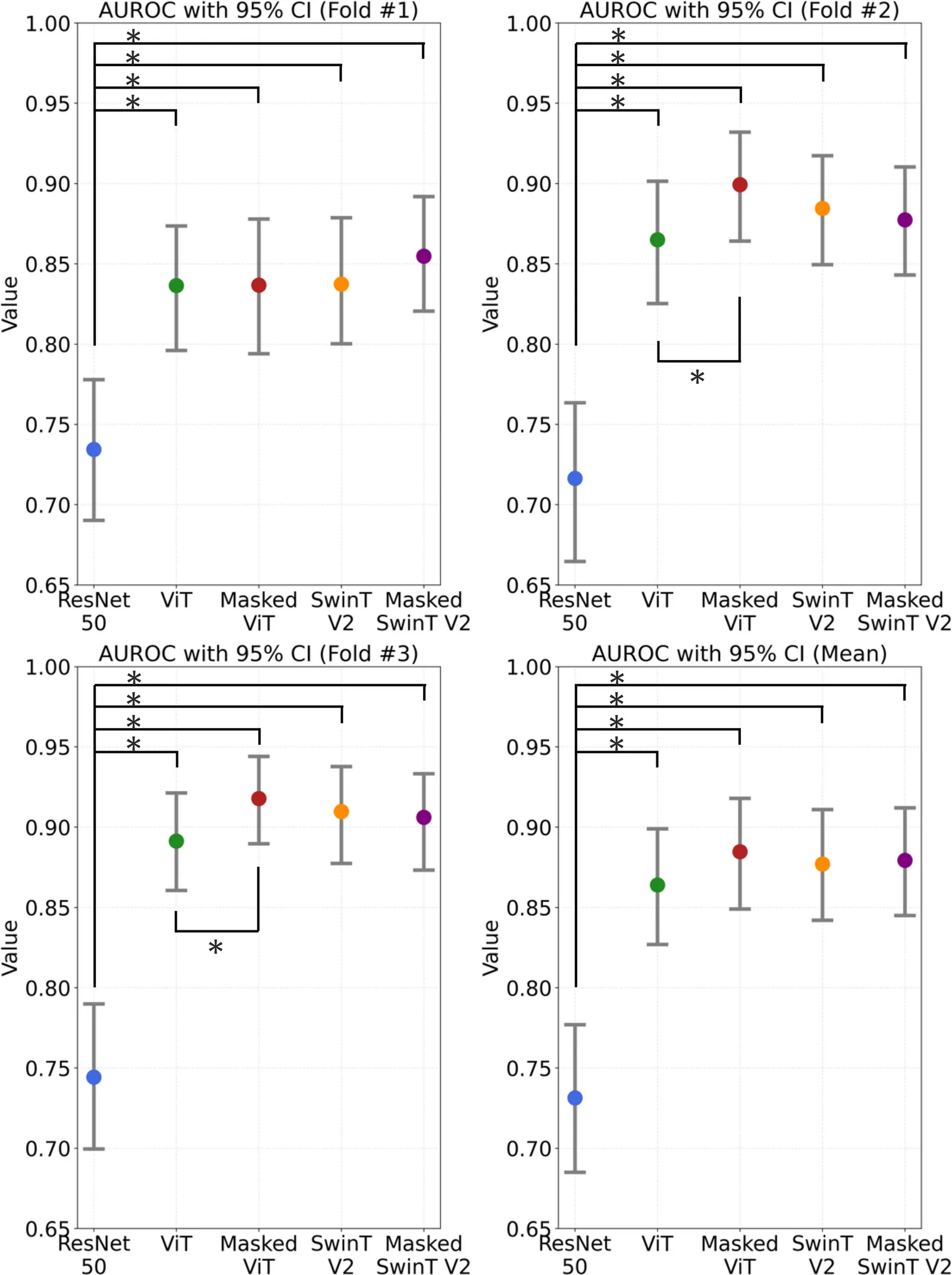
AUROC for each model across folds: The curves for each model are presented with 95% confidence intervals. Statistical comparisons between models for each fold were conducted using Delong test, and significant differences (p < 0.05) are indicated by asterisks (*).

Table 2 shows the performance values for each model, whereas Fig. 3 illustrates the AUROC values along with the statistical significance results. In Fold #1, the model with the highest AUROC was Masked SwinT V2 (AUROC = 0.885, AUPRC = 0.851, ACC = 0.795, SEN = 0.868, SPE = 0.704, F1 = 0.824, MCC = 0.584). The AUROC of ResNet50 was significantly lower than those of other models. In Fold #2, the model with the highest AUROC was Masked ViT (AUROC = 0.899, AUPRC = 0.893, ACC = 0.865, SEN = 0.926, SPE = 0.779, F1 = 0.890, MCC = 0.721). The AUROC of ResNet50 was significantly lower than those of other models, and AUROC of Masked ViT was significantly higher than that of conventional ViT. In Fold #3, the model with the highest AUROC was Masked ViT (AUROC = 0.918, AUPRC = 0.921, ACC = 0.857, SEN = 0.795, SPE = 0.934, F1 = 0.861, MCC = 0.726). The AUROC of ResNet50 was significantly lower than those of other models, and AUROC of Masked ViT was significantly higher than that of conventional ViT. Furthermore, despite the data imbalance between Normal and Benign within class 0, Masked ViT correctly predicted some Benign cases (Appendix 4). In summary, Masked ViT showed the highest mean AUROC across all three folds.

**Table 2:** Model comparison

Method	Fold #	AUROC [95% CI]	AUPRC [95% CI]	ACC	SEN	SPE	F1	MCC
**ResNet50** [28]	1	0.734 [0.690–0.778]	0.749 [0.691–0.813]	0.708	0.736	0.672	0.736	0.408
	2	0.716 [0.665–0.764]	0.760 [0.696–0.824]	0.635	0.459	0.884	0.596	0.363
	3	0.774 [0.700–0.790]	0.797 [0.743–0.848]	0.701	0.629	0.792	0.701	0.421
	Mean	0.731 [0.685–0.777]	0.769 [0.710–0.828]	0.681	0.608	0.783	0.678	0.397
**ViT** [17]	1	0.836 [0.796–0.874]	0.825 [0.771–0.882]	0.788	0.804	0.767	0.808	0.571
	2	0.865 [0.825–0.901]	0.879 [0.833–0.923]	0.827	0.906	0.715	0.860	0.641
	3	0.891 [0.861–0.921]	0.910 [0.877–0.939]	0.835	0.847	0.820	0.851	0.666
	Mean	0.864 [0.827–0.899]	0.871 [0.827–0.915]	0.817	0.852	0.767	0.840	0.626
**Masked ViT (ours)**	1	0.837 [0.794–0.878]	0.809 [0.746–0.876]	0.811	0.906	0.693	0.842	0.620
	2	0.899 [0.864–0.932]	0.893 [0.846–0.940]	0.865	0.926	0.779	0.890	0.721
	3	0.918 [0.890–0.944]	0.921 [0.878–0.956]	0.857	0.795	0.934	0.861	0.726
	Mean	0.885 [0.849–0.918]	0.874 [0.823–0.924]	0.844	0.876	0.802	0.864	0.689
**SwinT V2** [29]	1	0.837 [0.800–0.879]	0.822 [0.767–0.877]	0.792	0.906	0.651	0.829	0.584
	2	0.884 [0.849–0.917]	0.899 [0.861–0.933]	0.841	0.906	0.750	0.870	0.671
	3	0.910 [0.877–0.938]	0.918 [0.876–0.951]	0.857	0.886	0.820	0.873	0.709
	Mean	0.877 [0.842–0.911]	0.880 [0.835–0.920]	0.830	0.899	0.740	0.857	0.655
**Masked SwinT V2 (ours)**	1	0.855 [0.820–0.892]	0.851 [0.803–0.902]	0.795	0.868	0.704	0.824	0.584
	2	0.877 [0.843–0.910]	0.902 [0.866–0.935]	0.832	0.914	0.715	0.864	0.651
	3	0.906 [0.873–0.933]	0.918 [0.881–0.947]	0.867	0.913	0.809	0.884	0.729
	Mean	0.879 [0.845–0.912]	0.890 [0.850–0.928]	0.831	0.898	0.743	0.857	0.655

Abbreviations: 95% CI: 95% confidence interval; AUROC: area under the receiver-operating characteristic curve; AUPRC: area under the precision recall curve; ACC: accuracy; SEN: sensitivity; SPE: specificity; F1: F1-score; MCC: Matthews correlation coefficient

## Discussion

Our study aimed to evaluate the effectiveness of shape-guidance strategies by varying the combinations of encoder layers that guide attention to breast structures. Masked ViT and Masked SwinT V2 with masking at the Shallow and Deep stages achieved the highest AUROC among their respective models. In particular, Masked ViT achieved an AUROC of 0.885 [95% CI: 0.849–0.918], outperforming the conventional models.

Sun et al. constructed a Transformer model with Cross-View Attention Blocks using 3,564 mammography images from the Digital Database for Screening Mammography dataset for classifying images as cancer or non-cancer [35]. Their SwinT model applied the blocks at the final encoder stage and achieved an AUROC of 0.843 ± 0.004, outperforming the ResNet50-based model, which achieved 0.752 ± 0.003. You et al. developed a contrastive learning framework for the same classification task using Lesion and Normal Contrastive Losses and 88,753 mammography images from 10 institutions [36]. Their proposed loss function was able to better discriminate intra-class features, achieving an AUROC of 0.885 ± 0.009 compared to the baseline AUROC of 0.862 ± 0.010. Although a direct comparison is not feasible, our proposed model demonstrated comparable or even superior performance. From a clinical perspective, the average sensitivity of screening mammography in the U.S. is 86.9% [95% CI: 86.3–87.6%] and the average specificity is 88.9% [95% CI: 88.8–88.9%] [37]. Recent study also indicated that when adding AI support, radiologists achieved higher performance, with sensitivity of 87.2% and specificity of 91.1% [38]. Compared to these benchmarks, our model tended to show higher sensitivity than specificity.

Interestingly, our strategy was found to be more effective in ViT than in SwinT V2. In ViT, selecting the patch size involves a trade-off: larger patches capture global structures but may miss fine details, whereas smaller patches focus on local patterns while sacrificing broader contextual understanding [17]. SwinT models overcome this limitation through window-based attention and a hierarchical architecture, which enable effective multi-scale feature learning [29, 34]. Applying guidance to both the Shallow and Deep layers in ViT may have allowed it to capture both global and local features in a manner similar to SwinT V2. This may explain why Masked ViT outperformed SwinT V2 and Masked SwinT V2, suggesting that the shape-guided strategy and window mechanism may offer comparable advantages.

**Fig. 4:**
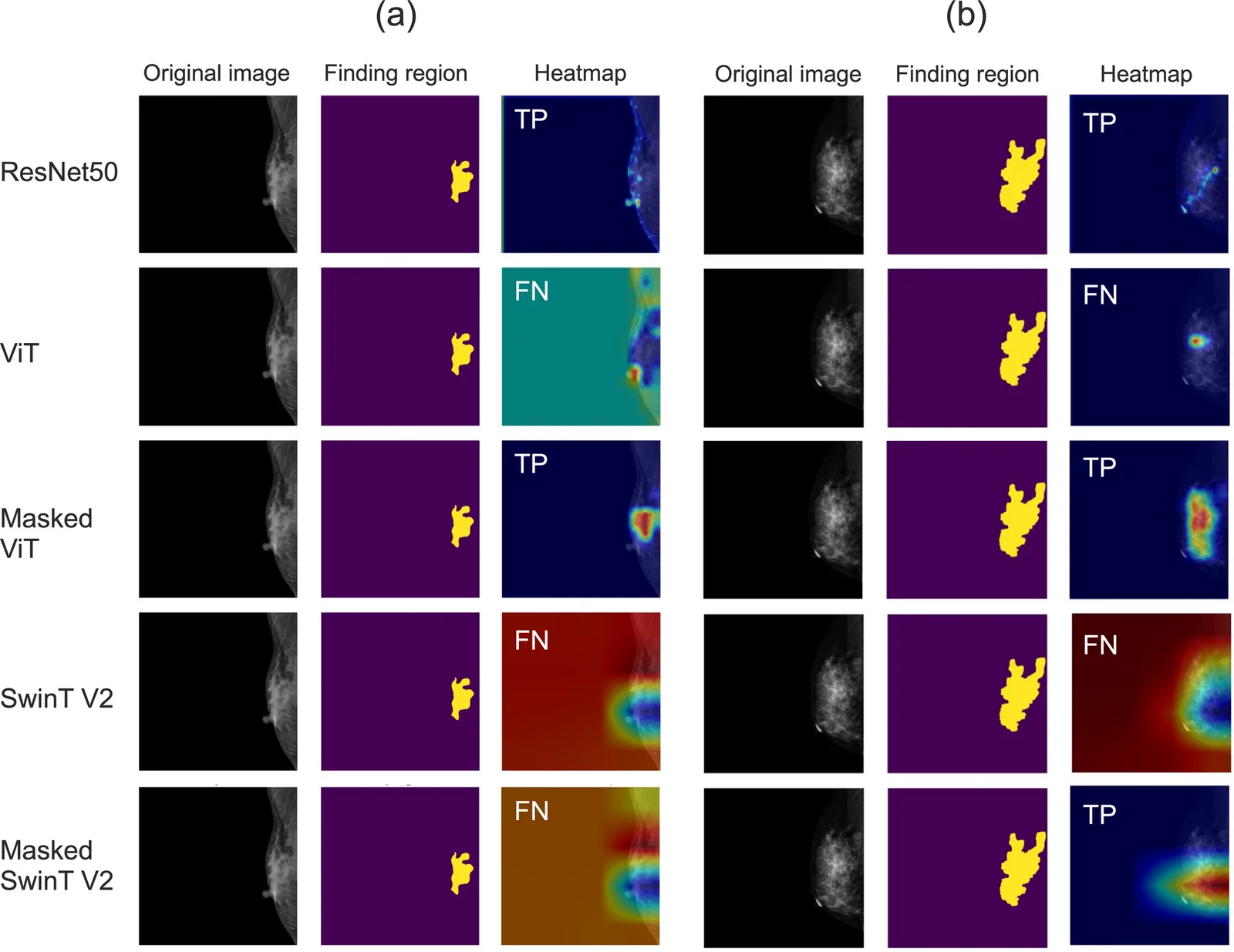
Grad-CAM visualizations for each model in cases with a single finding: Grad-CAM heatmaps based on predicted labels are shown for (**a**) a malignant mass, and (**b**) a malignant calcification. For each case, the first column displays the original image, the second presents the segmentation label of the finding, and the third shows the Grad-CAM heatmap overlaid on the original image. TP and FN indicate true positive and false negative, respectively.

**Fig. 5:**
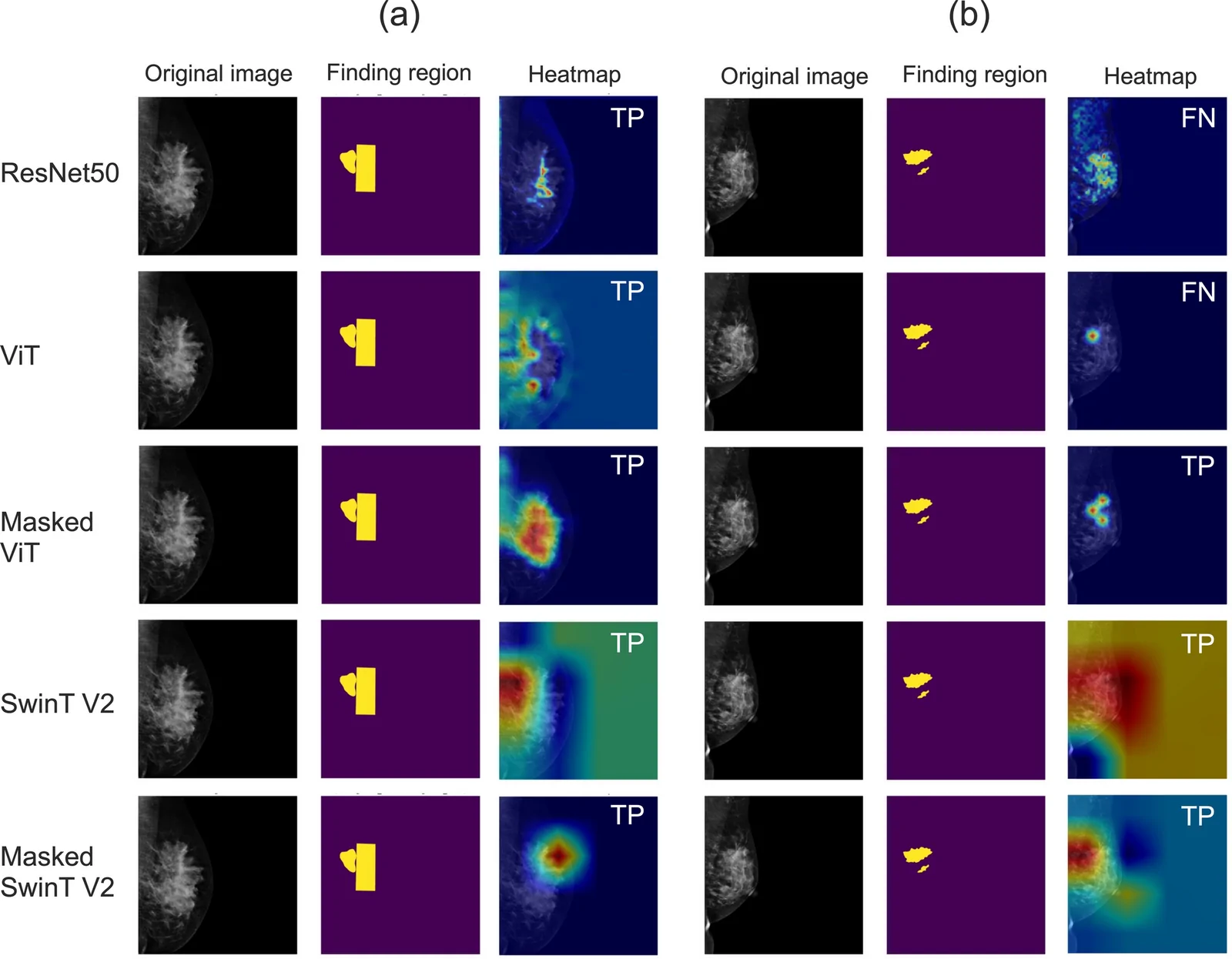
Grad-CAM visualizations for each model in cases with multiple findings: Grad-CAM heatmaps based on predicted labels are shown for (**a**) a malignant calcification with architectural distortion, and (**b**) a malignant mass with a malignant calcification. For each case, the first column displays the original image, the second presents the segmentation label of the finding, and the third shows the Grad-CAM heatmap overlaid on the original image. Bounding boxes were used to define architectural distortion, as the borders with the surrounding normal breast tissue are indistinct. TP and FN indicate true positive and false negative, respectively.

Heatmap visualization is considered essential for clinical implementation of Transformer-based models, although their interpretability remains under debate [39]. Figure 4 and Fig. 5 show gradient-weighted class activation mapping (Grad-CAM) for cases with single and multiple findings, respectively. ResNet50 focused mainly on local features, effectively capturing directional patterns corresponding to malignant calcifications. However, the distribution of the captured regions remained limited. In contrast, Transformer-based models covered relatively broader regions than ResNet50. In several cases, the shape-guided strategy enabled Masked ViT and Masked SwinT V2 to focus precisely on lesion regions. As an additional analysis, we measured the overlap and the coverage between model heatmaps and annotations in ViT and Masked ViT models (Appendix 5). The use of the shape-guided strategy led to a broader recognition area, which in turn resulted in more reliable coverage of the annotated regions. This suggests that mask-based guidance remains a valuable approach for improving the spatial attention of Transformer models.

Our study demonstrates that selective masking at only the Shallow and Deep layers was most effective for Transformer-based models. A previous study has shown that attention in shallow layers tends to capture local features, whereas the effect of preserving the original input is dominant especially in the deep layers [40], possibly because the deeper layers mix a relatively large amount of contextual or positional information into the [CLS] token [41]. Liu et al. modified the Transformer to enhance the input in residual connections, improving performance and convergence in language tasks [42]. In our study, individual masks in the shallow layers may enhance local features, while those in the deeper layers effectively transfer object-level positional information to the [CLS] token. When developing ViT-Large with 24 layers, Dosovitskiy et al. showed that the 12th and subsequent layers tended to have higher “mean attention distance” (i.e., attention spread) for all heads [17]. Although we used ViT-Base with 12 layers, the object-level enhancement by applying our strategy to the deep layers may be also explained from their study.

**Fig. 6:**
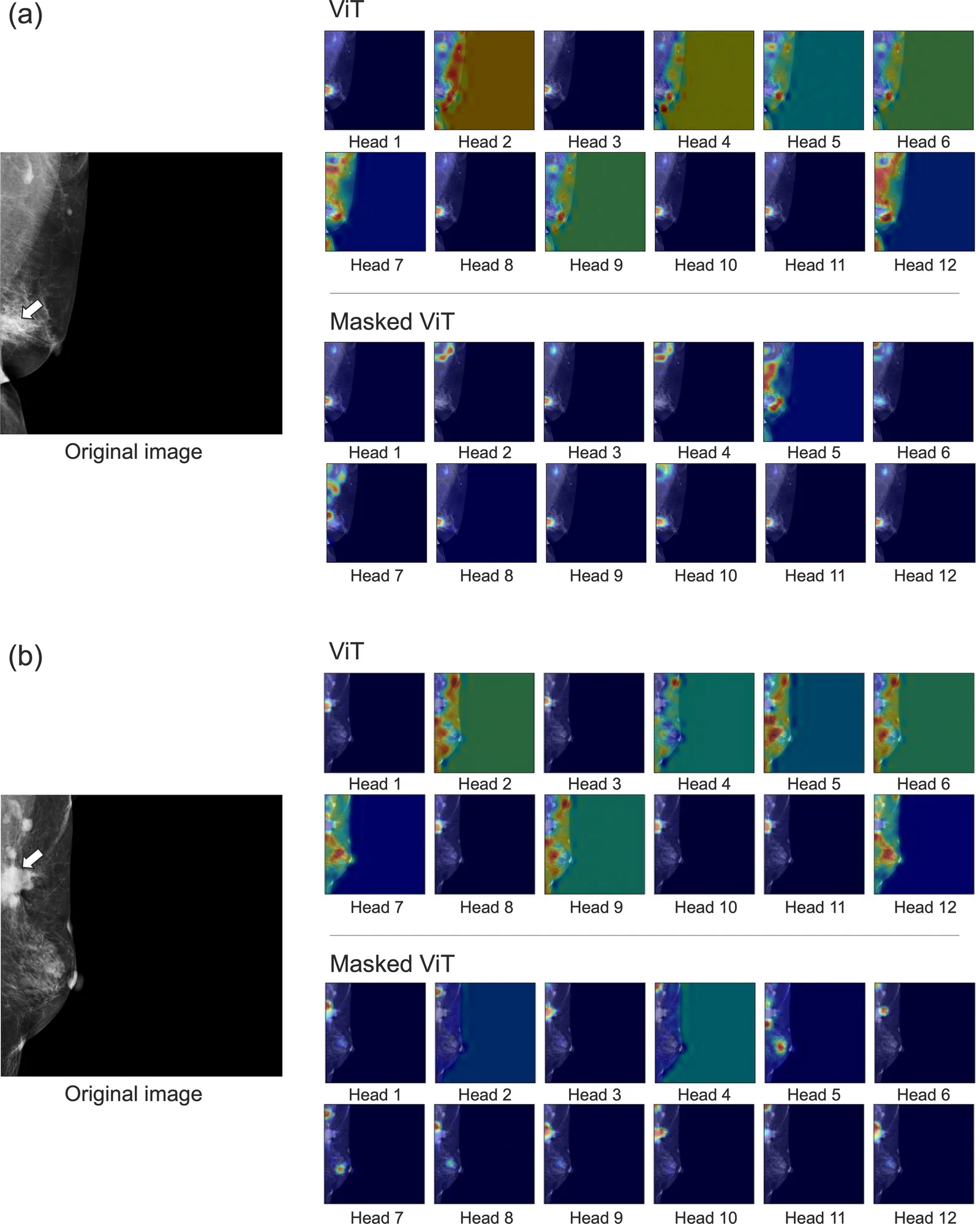
Attention map for each attention head: Attention heads are shown for cases of (**a**) malignant architectural distortion, and (**b**) malignant mass. Although some attention heads continued to partially attend to background regions, the guiding strategy resulted in a greater number of heads focusing on the breast region.

The effect of shape guidance was also observable in the attention heads (Fig. 6). While the strategy increased the attention on the breast region, not all the heads consistently attended to the breast region across all layers (Appendix 6). This suggests that multi-head self-attention is not constrained solely to the intended region, and some heads may retain fixed spatial preferences. A previous study by Kobayashi et al. [43] revealed that components in self-attention canceled out each other’s effects on Bidirectional Encoder Representations from Transformers (BERT) models. In addition, a recent study by Darcet et al. revealed that low-informative background areas of natural images are repurposed for internal computations in Transformer models [44]. We applied the same shape-guided strategy to all attention heads; however, future studies could optimize guidance by identifying specific roles for each attention head. A previous study has already analyzed selective roles of attention heads in language models [45], and similar investigations are needed in vision models as well [40]. As a preliminary step, we examined the interrelationships among attention heads by graph analysis (Appendix 7), inspired by DeRose et al., who used graphs to identify token dependencies in language models [46]. While quantitatively analyzing the interrelationships among attention heads could help clarify their selective roles, further work is needed to support detailed diagnostic reasoning.

Our study has some limitations. First, we used a single dataset, which may restrict generalizability. Exploring the generalizability across different institutions should be validated in future studies. Second, we focused only on MLO images. While MLO views have fewer blind spots compared to craniocaudal (CC) views [47], incorporating both views will be required for developing a comprehensive and generalizable model [48]. Third, we trained our models from scratch. Recently, various learning strategies such as self-supervised and weakly supervised learning have emerged alongside transfer learning [49, 50]. Although more computationally demanding, these learning strategies may enhance model performance. Finally, we did not evaluate all possible combinations of layers for mask application, but instead grouped them into three representative stages. Since the number of transformer layers is a tunable hyperparameter, our focus was on capturing general trends rather than identifying the impact of individual layers. A more fine-grained analysis is left for future work.

## Conclusions

We proposed Transformer-based models that guide attention to breast structures for predicting breast cancer in mammography. Applying shape-guided masking at both shallow and deep encoder layers improved model performance by enhancing morphological feature recognition. Although the application of Transformer models to mammography remains limited, it is expected to gain increasing importance in future research.

## Supplementary Information

Below is the link to the electronic supplementary material.

ESM 1 (10.5 MB DOCX)

## Abbreviations

ROI: Regions of interest
MLO: Mediolateral oblique
CC: Craniocaudal
CMMD: Chinese Mammography Database
CAD: Computer-aided diagnosis
AI: Artificial intelligence
ViT: Vision Transformer
SA: Self-attention
MHSA: Multi-head self-attention
MSA: Masked self-attention
SwinT: Swin Transformer
SwinT V2: Swin Transformer V2
CNN: Convolutional neural network
ResNet: Residual neural network
BERT: Bidirectional Encoder Representations from Transformers
GELU: Gaussian error linear unit
WCE: Weighted cross-entropy
AUROC: Area under the receiver-operating characteristic curve
AUPRC: Area under the precision recall curve
ACC: Accuracy
SEN: Sensitivity
SPE: Specificity
F1: F1-score
MCC: Matthews correlation coefficient
95% CI: 95% Confidence interval
Grad-CAM: Gradient-weighted class activation mapping
