# Supplementary material for: Transformer-based Deep Learning Models with Shape Guidance for Predicting Breast Cancer in Mammography Images
Source: J Imaging Inform Med. 2025 Dec 19;39(5):4038–51. doi: 10.1007/s10278-025-01773-3 (PMC13694632; doi:10.1007/s10278-025-01773-3)
Supplement: Supplementary file 1 [file 10278_2025_1773_MOESM1_ESM.docx]

**Supplementary Materials**

**Title:**

Transformer-based Deep Learning Models with Shape Guidance for Predicting Breast Cancer in Mammography Images

**Appendix 1.**

Fig 1. ROC curves at different image resolutions.

As a preliminary experiment, ROC curves are shown for various image resolutions (224×224, 512×512, and 1024×1024). The results showed a tendency for performance to decrease as the resolution decreased. The decline in diagnostic performance of AI models with lower-resolution mammography images was also suggested in a previous study [1].

**Appendix 2.**

Fig 2. Image preprocessing

The original images (2294 × 1914 pixels) were padded with zeros on the background side opposite the breast to form square images of 2294 × 2294 pixels. The red box denotes the padded region. Images were then resized to 2048 × 2048 pixels using LANCZOS resampling with the PILLOW library. LANCZOS resampling is an interpolation method that uses a sinc filter to minimize aliasing and preserve fine details when resizing images.

**Appendix 3.**

Fig 3. ROC and PR curves for SwinT and SwinT V2.

The mean ROC and PR curves across folds for SwinT and SwinT V2 are presented. The results indicated that SwinT V2 was more effective for learning high-resolution images. This finding is consistent with previous studies showing that SwinT V2 enables stable training even with high-resolution inputs and large-scale datasets [2]. This improved performance can be attributed to architectural modifications in SwinT V2, which introduces scaled cosine attention and post-norm layers to the original SwinT. These modifications help prevent training saturation and support stable optimization even when handling high-resolution images.

**Appendix 4.**

Fig 4. Number of correct prediction cases for Normal and Benign in negative class.

This figure illustrates the number of correct prediction cases for Normal and Benign among negative class (solid bars) and the total number of samples (transparent bars) in Masked ViT model. Despite the data imbalance between Normal and Benign within negative class, the model correctly predicted some Benign cases as well. On the other hand, more than half of the Benign cases were misclassified as malignant, particularly in Fold 1 and Fold 2. These results imply that classifying malignant and benign cases remains a challenge for the Transformer models.

**Appendix 5.**

Fig 5-1. Consistency measurement.

Grad-CAM heatmaps were generated from original images based on positive model predictions for malignant cases. A saliency map ($S$) was generated using the threshold for each image. The threshold was calculated as the sum of the mean and standard deviation of the Grad-CAM values for each image. To assess the consistency between the saliency map and annotation ($G$), the overlapping region ($G\cap S$) is computed. Two quantitative metrics are derived:

$$Ground Truth Cover \left( GC \right)= \frac{\left| G\cap S \right|}{\left| G \right|},(1)$$

$$Saliency Cover \left( SC \right)= \frac{\left| G\cap S \right|}{\left| S \right|},(2)$$

where $GC$ represents the proportion of the annotated area captured by the AI, and $SC$ indicates the proportion of the area corresponding to the annotation (See Fig. 5-1). Statistical comparison was performed using the Wilcoxon Signed-Rank Test, with statistical significance defined as a p-value < 0.05.

As shown in Fig. 5-2, guidance led to an increase in GC from 0.595 [95% confidence interval: 0.551–0.639] to 0.692 [0.642–0.741], and a decrease in SC from 0.190 [0.160–0.221] to 0.138 [0.114–0.162]. These results indicate, at least, that the use

Fig 5-2. Comparison of SC and GC with and without guidance.

of mask guidance allowed the ViT model to capture a broader area corresponding to malignant findings. In addition, GC values were higher than SC in both models, suggesting that the highly activated saliency maps captured by ViT tended to cover a wider contextual area. This result can be attributed to the ability of the Transformer architecture to capture global features.

**Appendix 6.**

Fig 5. Attention heads across layers in masked and non-masked ViT.

This figure shows the attention maps from each head at each layer of the non-masked and masked ViT models. While applying the mask reduced the number of attention heads attending to background regions, not all attention heads consistently attended to the breast region across all images. Recent studies have explored quantitative methods for analyzing the roles of individual attention heads in ViT [3, 4]. In Appendix 7, we present a methodology for such quantitative analysis using graph-based approaches.

**Appendix 7.**

Fig 7-1. Overlap rate computation for attention head analysis.

There is limited understanding of the behavior of attention heads in ViTs. Previous work in language models has identified heads responsible for encoding positional or syntactic information [3]. Interpreting the attention heads in ViTs is essential for providing detailed reasoning behind diagnostic decision-making. Li et al. identified significant heads on natural images using leave-one-out ablation [4] However, their method overlooks head interactions, highlighting the need for another approach for exploring their relationships. Inspired by DeRose et al., who used graphs to identify token dependencies in language models [5], we introduce a method to represent the interrelationships among attention heads by graph analysis. The suggested analysis captures the distribution trends of attention heads across the entire dataset and enables visualization of how the interrelationships between heads vary with shape-guided strategies.

We identified the attention heads that contributed to decision-making of the ViT model based on the morphological features of the breast. Two types of overlap rate (*O*) were calculated between the two visual components (See Fig. 7-1). After normalizing the attention heads of the last layer to create a one-hot image with a threshold of 0.5, we calculated the overlap rate matrices for all combinations (See Fig. 7-2). Note that although normalizing all attention heads ignores the absolute importance of individual heads, our aim is to analyze interrelationships among heads. The calculation method is inspired by the Shared Interest method [6], which identifies patterns in model behavior quantitatively. The *O* matrix, averaged over the dataset, is represented as a directed graph, a mathematical structure for capturing relationships between vertexes [7]. Vertexes were aggregated into “communities” by the Louvain method [8], which detects communities in graphs based on modularity $Q$ maximization. For a directed graph with

Fig 7-2. Overview for graph-based association analysis.

*m* edges, the modularity gain $\Delta Q$ can be calculated [9] as:

$$\Delta Q= \frac{k_{i,in}}{m}- \gamma\frac{k_{i}^{out} \cdot\sum_{out}^{in} + k_{i}^{in} \cdot\sum_{in}^{out}}{m^{2}},(3)$$

where $k_{i}^{out}, k_{i}^{in}$ are the inner and outer weighted degrees of node *i*, and $\sum_{tot}^{in} , \sum_{tot}^{out}$are the sum of in-going and out-going edges incident to vertexes in a community *C*. $\gamma$ is the resolution parameter, where values less than 1 favor larger communities, and values greater than 1 favor smaller ones. We set the parameter to the default value of 1. The Louvain method was implemented using the NetworkX package (version 3.4.2).

Graph from mean overlap rate matrix enables to visualize head tendencies across the true-positive cases, without showing all slices for all patients (See Fig. 7-3). The shape-guided strategy tended to increase the number of attention heads focusing on breast structures, with different heads attending to distinct regions within the breast.

Fig 7-3. Directed graph for each model.

**References**

1. Inamori R, Kaneno T, Oba K, Takaya E, Hirahara D, Kobayashi T, Kawaguchi K, Adachi M, Shimokawa D, Takahashi K, Tsunoda H, Ueda T: Impact of Downsampling Size and Interpretation Methods on Diagnostic Accuracy in Deep Learning Model for Breast Cancer Using Digital Breast Tomosynthesis Images. Tohoku J Exp Med 6;265(1):1-6, 2025
2. Liu Z, Hu H, Lin Y, Yao Z, Xie Z, Wei Y, Guo J, Zhang L, Dai Q, Yuan L, Bao J: Swin Transformer V2: Scaling Up Capacity and Resolution. Proceedings of the IEEE/CVF Conference on Computer Vision and Pattern Recognition (CVPR) 11999–12009, 2022
3. Voita E, Talbot D, Moiseev F, Sennrich R, Titov I: Analyzing Multi-Head Self-Attention: Specialized Heads Do the Heavy Lifting, the Rest Can Be Pruned. arXiv preprint arXiv:1905.09418, 2019
4. Li Y, Wang J, Dai X, Wang L, Yeh CM, Zheng Y, Zhang W, Ma KL: How Does Attention Work in Vision Transformers? A Visual Analytics Attempt. IEEE Trans Vis Comput Graph 29(6):2888-2900, 2023
5. DeRose JF, Wang J, Berger M: Attention Flows: Analyzing and Comparing Attention Mechanisms in Language Models. IEEE Trans Vis Comput Graph 27(2):1160-1170, 2021
6. Boggust A, Hoover B, Satyanarayan A, Strobelt H: Shared Interest: Measuring Human-AI Alignment to Identify Recurring Patterns in Model Behavior. Proceedings of the 2022 CHI Conference on Human Factors in Computing Systems 10:1–17, 2022
7. Gori M, Monfardini G, Scarselli F: A new model for learning in graph domains. Proceedings of the 2005 IEEE International Joint Conference on Neural Networks (IJCNN) 2:729–734, 2005
8. Blondel VD, Guillaume JL, Lambiotte R, Lefebvre E. Fast unfolding of communities in large networks. J Stat Mech Theory Exp 10008, 2008
9. Dugué N, Perez A: Directed Louvain: maximizing modularity in directed networks. Université d’Orléans, 2015
